# Selenium Nanoparticles Regulate Antioxidant Enzymes and Flavonoid Compounds in *Fagopyrum dibotrys*

**DOI:** 10.3390/plants13213098

**Published:** 2024-11-03

**Authors:** Ting Hu, Sasa Zhang, Kui Li, Yanbin Guo

**Affiliations:** 1 College of Life Science, Anqing Normal University, Anqing 246000, China; 2 Collaborative Innovation Center of Targeted Development of Medicinal Resources, Anqing 246000, China; 3 College of Resources and Environmental Sciences, China Agricultural University, Beijing 100193, China; zhangsa1992@126.com (S.Z.); likui9090@163.com (K.L.)

**Keywords:** selenium nanoparticles, uptake, transformation, *Fagopyrum dibotrys*, flavonoid

## Abstract

*Fagopyrum dibotrys* is a herbal plant. Selenium (Se) is a beneficial element for plants; selenium nanoparticles (SeNPs) are gaining importance in food and agriculture due to their low toxicity and high activity. This study revealed that foliar application of SeNPs enhanced superoxide dismutase, glutathione peroxidase, and peroxisome activities and significantly enhanced the flavonoid compound content in *F. dibotrys*. SeNPs with a concentration of 5.0 mg L^−1^ also promoted the growth of *F. dibotrys*. The foliar application of SeNPs could be absorbed by pores in leaves of *F. dibotrys* and mainly transformed to selenomethionine (32.5–43.2%) and selenocysteine (23.4–38.4%) in leaves and tubers of *F. dibotrys.* Consequently, this study offers a profound understanding of plants’ uptake and biotransformation of SeNPs. Furthermore, the findings of this study have suggested that SeNPs can be applied to improve the quantity and quality of the herbal plant of *F. dibotrys*.

## 1. Introduction

Selenium is a vital micro-nutrient element for humans, animals, and microbes, and it is also helpful in plants [1,2]. Generally, appropriate concentrations of Se levels promote plant growth and enhance stress tolerance, while excessive Se concentrations can result in toxicity [2,3]. Selenium functions as an antioxidant by mitigating the buildup of free radicals or reactive oxygen species (ROS), thereby averting oxidative stress [4,5]. It also leads to the stimulation of photosynthetic pigment creation, net photosynthetic ratio, gas exchange, and the production of secondary metabolites during the process of photosynthesis [2,6,7]. High levels of Se may cause adverse effects and act as a pro-oxidant, which promotes the formation of lipid peroxidation byproducts and subsequently leads to significant reductions in crop yields [8,9]. Therefore, Se’s application in plant nutrition and physiology remains an interesting topic. Lately, there has been an enormous increase in the prospective utilization of nanotechnology within the domains of food and agriculture. Selenium nanoparticles (SeNPs) are a nanostructured manifestation of Se that can be manufactured by chemical and biosynthetic techniques [10,11]. SeNPs have garnered significant attention owing to their numerous advantages, which encompass chemical stability, biocompatibility, and minimal toxicity [11,12]. Our previous research found that SeNPs can be absorbed and transformed into selenomethionine and selenocystine by wheat and rice root [13,14]. In previous studies, it was observed that ZnO-NPs have the ability to traverse the surface of wheat leaves by utilizing the stomata as entry points. Subsequently, these nanoparticles are dispersed throughout the mesophyll tissue and can be taken up by the leaf tissue [15,16]. Nevertheless, few studies have investigated the transformation and translocation of absorbed foliar-applied SeNPs in plants.

*Fagopyrum dibotrys*, or golden buckwheat, is a perennial herbaceous plant with an upright growth habit belonging to the Fagopyrum genus within the Polygonaceae family [17]. It predominantly thrives in the northern temperate zone and has a vast distribution throughout many regions like China, Kazakhstan, Russia, etc. [18,19]. Extracts from *F. dibotrys* have antioxidant, anti-inflammatory, and anti-cancer properties [19,20,21], making it a potential candidate for natural remedies and supplements. *F. dibotrys* is also popular as a functional food due to its nutritional content, especially its seeds, which are rich in protein, fiber, minerals, and fatty acids [19]. Previous research studies have revealed the existence of flavonoid and phenolic chemicals within the entirety of the *F. dibotrys* plant [21,22,23]. Flavonoids are synthesized through the phenylpropanoid pathway [24], and phenylpropanoid compounds are induced in response to biotic or abiotic stresses [20,25,26]. Selenium’s antioxidant properties have been well documented, and its incorporation into medicinal plants could enhance their therapeutic effects [27]. For example, combining Se with the polysaccharides from *Astragalus membranaceus* has improved immune function and protected against oxidative stress [28]. As previously stated, the presence of Se may act as a trigger for the biosynthesis of flavonoid compounds in plants. Investigating SeNPs’ utilization in medicinal herbs can enhance our comprehension of plant Se accumulation and resistance mechanisms. Additionally, this research may facilitate the development of Se-enriched products with enhanced nutritional value and improved quality. Therefore, the main objective of our research is to (1) analyze the capacity of *F. dibotrys* to absorb, accumulate, and transform SeNPs and (2) evaluate the effects of SeNPs on the growth, antioxidant enzymes, and flavonoid contents of *F. dibotrys*.

## 2. Materials and Methods

### 2.1. SeNP Preparation and Characterization

The SeNPs were synthesized via a chemical approach by adding varying amounts of sodium thiosulfate (Na_2_S_2_O_3_) to a redox system of sodium selenite (Na_2_SeO_3_) and sodium dodecyl sulfate (SDS) as previously described [29]. Briefly, SeNPs were synthesized from Na_2_SeO_3_ with Na_2_S_2_O_3_ as the reducing agent and SDS as a surfactant stabilizer. The SeNPs were collected by centrifugation, washed three times with 10 mM SDS, and suspended with deionized water. The size and morphology of the synthesized SeNPs were analyzed using a transmission electron microscope (TEM) (HT-7700 TEM, Hitachi Co., Tokyo, Japan) with energy-dispersive X-ray spectroscopy (EDS) and employed to examine the elemental composition of SeNPs.

### 2.2. Plant Materials, Growing Conditions, and Experimental Design

The tubers of *F. dibotrys* were provided by the Key Laboratory of Hunan Forest and Chemical Industry Engineering and identified by Prof. C. G. Xiang, Ji Shou University. Each pot was filled with 10.0 kg of dried soil collected from an experimental field at China Agricuritural University; the pH was 6.8 and the total Se content was 0.18 mg/kg, with triplicate plants for each soil sample. Before filling the pots, the soil in each pot had been modified to reach a moisture level equivalent to 60% of its field water retention capacity using deionized water. The tubers of *F. dibotrys* were cut to the same size (4–6 cm) and then cultivated in a pot. *F. dibotrys* were grown in a greenhouse with 20 to 25 °C temperature, 60–70% humidity, and a 16/8 light/dark regime. SeNPs were added to deionized water and distributed well up to 0.5 mg L^−1^, 2.5 mg L^−1^, 5.0 mg L^−1^, 10.0 mg L^−1^, and 20.0 mg L^−1^, respectively. The pH of the Se solutions was adjusted to 6.5 ± 0.2. The final volume of the Se solution applied was 1 L per plot (5.23 m^2^). Spraying SeNPs during watering can be carried out in two phases: phase one during *F. dibotrys* flowering (90 days after cultivation) and phase two in the *F. dibotrys* grain-filling stage (128 days after cultivation). The control treatment received only deionized water. *F. dibotrys* was harvested after 158 days of cultivation. Then, the leaves, stalks, and tubers of *F. dibotrys* were washed with deionized water to remove soil particles and dried at 70 °C for 72 h. The dry matter of leaves, stalks, and tubers per pot was weighed and milled for further Se analysis.

### 2.3. Scanning Electron Microscope with Energy Dispersion Spectra Analysis (SEM-EDS)

After eight hours of SeNP foliar application, the leaves of *F. dibotrys* were collected and washed three times using sterilized Milli-Q water. The leaves were pre-treated with 4% glutaraldehyde for 24 h at 4 °C, followed by washing in 0.1 M sodium cacodylate buffer thrice at an interval of 15 min. The dehydration process was conducted using acetone at various concentrations (30%, 50%, 70%, 80%, 90%, and 100%). Subsequently, the dehydrated samples were affixed onto brass stubs and underwent gold coating prior to examination. The samples were subsequently observed using a scanning electron microscope (SEM), the Hitachi S-3400N model, with an accelerating voltage of 15 kV in secondary electron (SE) mode. Electron probe micro-analysis was carried out using SEM facilities for different precipitated particles on the sorbent surface. Energy dispersion spectra (EDS) were analyzed using an INCA Penta FETX3 combined with an SEM (JEOL-JSM-6360; JEOL, Tokyo, Japan).

### 2.4. Determination of Total Selenium

The content of Se in the treated tissues of *F. dibotrys* was measured using a hydride generation flame atomic fluorescence spectrometer (HG-AFS) (Beijing Jitian Analysis Instruction Co., located in Beijing, China). The samples, consisting of 250 mg, were subjected to digestion using 8 mL of 15.3 M HNO_3_ following the microwave digestion procedure described by Baldwin et al. [30]. Following the process of chilling, a volume of 2.5 mL of a 6 M hydrochloric acid (HCl) solution was introduced into each tube. Subsequently, the contents of the tubes were subjected to a temperature of 100 °C for one hour to reduce selenate to selenite, as described by Zhang et al. [31]. After cooling, the digestion solution was rinsed with water to 25 mL. The blank test and recovery test were both conducted simultaneously. HG-AFS determined the total Se content of the digestion solution. Blanks and a certified reference material (GBW 10014-Chinese cabbage, National Research Center for Certified Reference Material, Beijing) were included in each batch of samples for quality control. The recovery for GBW-10014 was 80.4–108.4%.

The transfer factor (TF) is the ratio of the element concentration in the plant tissue and soil [32]. The TF was utilized to quantify the capacity of plants to transport and accumulate Se in various plant tissues and calculated as follows [33]:

(1) $$TF\;leaf=\frac{Se\;concentration\times leaf\;biomass}{Se\;concentration\times volume}$$

(2) $$TF\;stalk=\frac{Se\;concentration\times stalk\;biomass}{Se\;concentration\times volume}$$

(3) $$TF\;tuber=\frac{Se\;concentration\times tuber\;biomass}{Se\;concentration\times volume}$$

### 2.5. Determination of Selenium Species

A powdered sample (250 mg) was combined with 5 mL of a protease XIV solution (8 mg L^−1^ concentration, obtained from Sigma Chemical Co., St. Louis, MO, USA). The mixture was then agitated for 24 h at a temperature of 37 °C. The supernatant was centrifuged at 5000× *g* for 15 min and filtered through 0.22 μm mixed cellulose nitrate filters. The identification of Se species in *F. dibotrys* was accomplished through the utilization of anion exchange chromatography (specifically, Hamilton PRP-X100, Switzerland) in combination with the ICP-MS detection system (HPLC-ICP-MS; Agilent LC 1260 series and ICP-MS 7700; Agilent Technologies, Santa Clara, CA, USA). The 100 µL samples were injected into an HPLC-ICP-MS system and separated using a 30-millimolar (NH_4_)_2_HPO_4_ solution at 1 mL min^−1^. The pH of the eluent was adjusted to 6.0. The identification of peaks was conducted based on the retention periods of standard compounds, namely selenocystine (SeCys_2_), selenite (Se(IV)), selenomethionine (SeMet), and selenate (Se(VI)), which were obtained from the National Research Center for Certified Reference Materials in Beijing, China. The detected Se species were quantified by utilizing the peak areas of the calibration curves through an HPLC workstation.

### 2.6. Determination of Chlorophyll and Carotenoid Concentrations

The freshly obtained leaf sample of *F. dibotrys* was sectioned into smaller pieces. A 100 mg portion of the sample was then deposited into a centrifuge tube with a 10 mL volume containing 95% ethyl alcohol. Subsequently, the centrifuge tube was promptly placed in a room devoid of light. Twelve hours later, chlorophyll a and b were measured at wavelengths 665 nm and 649 nm, and carotenoids were measured at 470 nm, by the UV-422G spectrophotometer [34].

Calculating the chlorophyll a and b amounts was performed using the following formulas:

(4) $$Chlorophyll\;a=\frac{\left(13.95A665-6.8A649)({mgL}^{-1}\right)}{freshweight(g)}$$

(5) $$Chlorophyll\;b=\frac{\left(24.96A649-7.32A665)({mgL}^{-1}\right)}{freshweight(g)}$$

(6) Total chlorophyll=chlorophyll a+chlorophyll b

### 2.7. Determination of Total Flavonoid Content

The total flavonoid content was determined on extracts from the leaves, stalks, and tubers of *F. dibotrys* by aqueous ethanolic solution (50% ethanol) upon heating in a boiling water bath for 90 min, with three replicates [35]. The total flavonoid content was measured according to Shraim et al. with some modifications [36]. The methanolic extract, measuring 250 μL, was combined with 1.25 mL of distilled water and 75 μL NaNO_2_ solution with a concentration of 50 mg mL^−1^. After 6 min, 150 μL 100 mg mL^−1^ AlCl_3_ was added, and the mixture was allowed to stand for 6 min. Then, 4 mL of 40 g L^−1^ NaOH and 2.5 mL of deionized water were added. The absorbance was measured at 510 nm by a UV-Vis double-beam Hitachi U-3010 spectrophotometer (Hitachi, Kyoto, Japan), and the total flavonoid content was expressed as mg g^−1^ rutin.

### 2.8. Determination of Enzyme Activity

Peroxidase (POD), superoxide dismutase (SOD), and glutathione peroxidase (GSH-Px) activity were measured to assess the influence of SeNPs on the antioxidant system of *F. dibotrys* exposed to various treatments. Fresh leaves (500 mg) were homogenized in liquid nitrogen, and then 5 mL 0.01 mol L^−1^ PBS buffer solution (pH 7.0) was added. The homogenate supernatant was separated by centrifuging at 10,000× *g* for 10 min. Activities of GPX-SH, SOD, and POD, as well as the protein quantification of tissues, were determined according to the method by Li et al. using assay kits from Nanjing Jiancheng Bioengineering Institute, Nanjing, China [37].

### 2.9. Statistical Analysis

The experiment was conducted using a randomized block design. The values in question denote the average measurements obtained from three distinct and separate trials conducted at different points in time. The collected data underwent a two-way analysis of variance (ANOVA) with a confidence level of 95%. The Duncan test was employed to determine the significance of differences between the mean values.

## 3. Results

### 3.1. SeNPs Increase the Biomass of F. dibotrys

**Figure 1:**
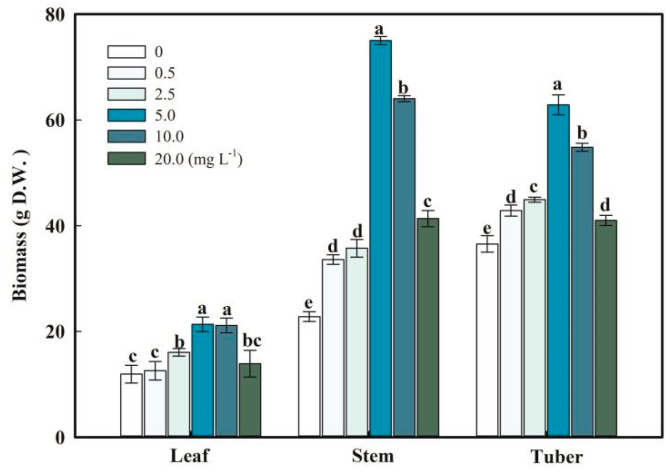
Biomass of *F. dibotrys.* Note: D.W. = dry weight. Data are presented as means ± SD (n = 3). Different letters in the same row represent significant differences between diets at the significance level of 0.05 (*p* < 0.05). See the text for a detailed description of the statistical methods of ANOVA and multiple comparison procedures.

The diameter of nanoparticles supplied in this experiment ranges between 81 and 152 nm (Figure S1A). The formation of nanoparticles was confirmed from the energy-dispersive X-ray spectroscopy. Se nanoparticles were identified from their characteristic absorption peaks at 1.37 KeV, 11.22 KeV, and 12.49 KeV (Figure S1B). After 158 days of experimentation, the leaf, stalk, and tuber were harvested under different treatments. The largest leaf biomass was observed in the 5.0 and 10.0 mg L^−1^ SeNP treatments, with a mean biomass of 21.34 ± 1.39 and 21.13 ± 1.40 g per pot, respectively. Similarly, giant stalk and tuber biomass was observed at 5.0 mg L^−1^, with a mean biomass of 75.01 ± 0.78 and 62.88 ± 1.90 g per pot, respectively (Figure 1).

### 3.2. Selenium Accumulation and Translocation in F. dibotrys

**Figure 2:**
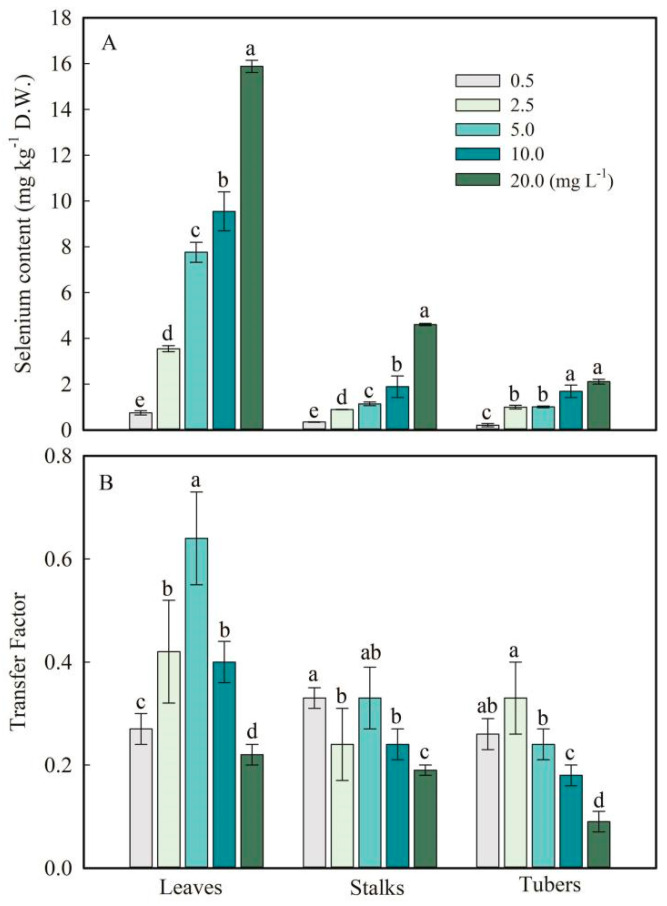
Selenium concentrations (**A**) and transfer factor (**B**) in leaves, stalks, and tubers of *F. dibotrys*. Values are mean ± SD; different letters above bars represent a significant difference (*p* < 0.05).

The average contents of Se in non-SeNP-treated tissues were 0.01 mg kg^−1^, 0.01 mg kg^−1^, and 0.05 mg kg^−1^ in the leaf, stalk, and tuber, respectively. Application of SeNPs significantly increased Se content in leaves, stalks, and tubers (Figure 2A). Under 0.5 to 20.0 mg L^−1^ SeNP foliar application, Se contents in leaves ranged from 0.75 ± 0.09 to 15.88 ± 0.27 mg kg^−1^; that in the stalks ranged from 0.35 ± 0.01 to 4.6 ± 0.05 mg kg^−1^; that in the tubers ranged from 0.21 ± 0.07 to 2.11 ± 0.11 mg kg^−1^, respectively (Figure 2A). The results indicate that *F. dibotrys* could uptake SeNPs and then transport them to the aerial parts successfully. Besides that, the Se content in the leaves was higher than in the stalks and tubers (Figure 2A).

In this work, the *F. dibotrys* tissue was divided into three parts, and the TF of Se from leaves, stalks, and tubers was calculated and is shown in Figure 2B. In general, Se transport from leaves to stalks and then to tubers highly depended on the concentration of SeNPs supplied. The TF values for *F. dibotrys* leaves, stalks, and tubers varied from 0.14 to 0.40, 0.12 to 0.23, and 0.05 to 0.21, respectively, when supplied with SeNP treatments from 0.5 to 20.0 mg L^−1^ (Figure 2B). In leaves and stalks, the maximum TF was attained when SeNPs were added at 5.0 mg L^−1^. At the same time, in tubers, the maximum TF was attained when SeNPs were added at 2.5 mg L^−1^ (Figure 2B). Moreover, as the Se dose increased in the foliar application, the TF in leaves tended to increase too, particularly in treatments with 0.5 to 5.0 mg L^−1^ SeNPs.

### 3.3. Selenium Species in F. dibotrys

**Figure 3:**
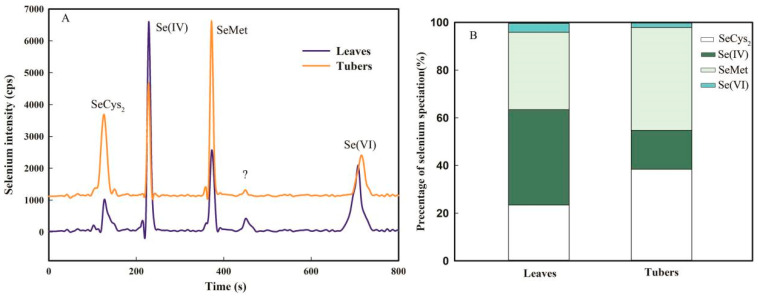
Chromatograms (**A**) and percentage of Se species (**B**) in leaves and tubers of *F. dibotrys* treated with 20.0 mg L^−1^ selenium nanoparticles. SeCys_2_, Selenocystine; Se (IV), selenite; SeMet, selenomethionine; Se(VI), selenate; ?, unknown species.

The enzymatic extraction procedure extracted 70.2–83.6% of the total Se from the leaves and tubers of *F. dibotrys*. Four main Se species were observed in *F. dibotrys*, including SeCys_2_, Se(IV), SeMet, Se(VI), and small levels of unknown Se species. Overall, the Se species percentage in leaves and tubers was different in *F. dibotrys* (Figure 3). In the leaves, the percentage of SeCys_2_, SeMet, and Se(IV) was 23.4 ± 1.3%, 32.5 ± 1.5%, and 40.4 ± 1.3%, respectively, and a small amount of Se(VI) (3.7 ± 0.8%) was also detected. Nevertheless, in the tubers, the percentage of organic Se species (SeCys_2_ occupied 38.4 ± 1.8%; SeMet occupied 43.2 ± 2.8%) reached up to 81.6%, while Se(IV) and Se(VI) occupied 16.3 ± 1.3% and 2.1 ± 0.5%, respectively (Figure 3B).

### 3.4. Selenium Effects on Flavonoid Contents in F. dibotrys

**Figure 4:**
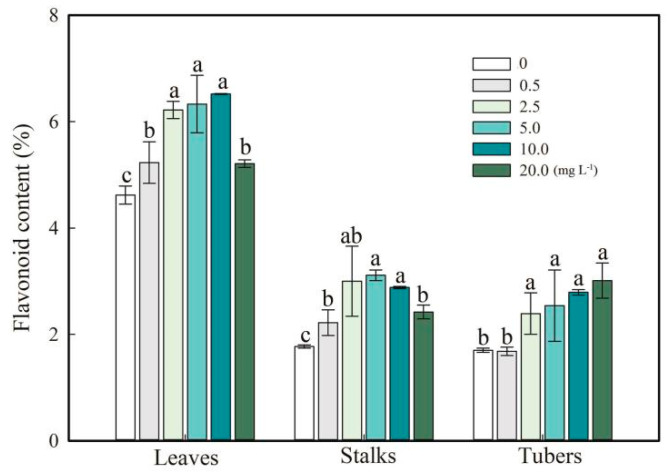
Flavonoid content in leaves, stalks, and tubers of *F. dibotrys.* Values are mean ± SD; different letters above bars represent a significant difference (*p* < 0.05).

Notably, an increase in flavonoid content was observed in *F. dibotrys* tissues supplemented with different amounts of SeNPs (Figure 4). The results show that the highest flavonoid content in leaves (6.2–6.5%) and stalks (2.9–3.0%) was obtained at 2.5 to 10.0 mg L^−1^ SeNP foliar fertilization. The highest flavonoid content in tubers (2.5–3.0%) was obtained with the 5.0–20.0 mg L^−1^ SeNP treatment (Figure 4).

### 3.5. Selenium Effects on Pigment Content in F. dibotrys

**Figure 5:**
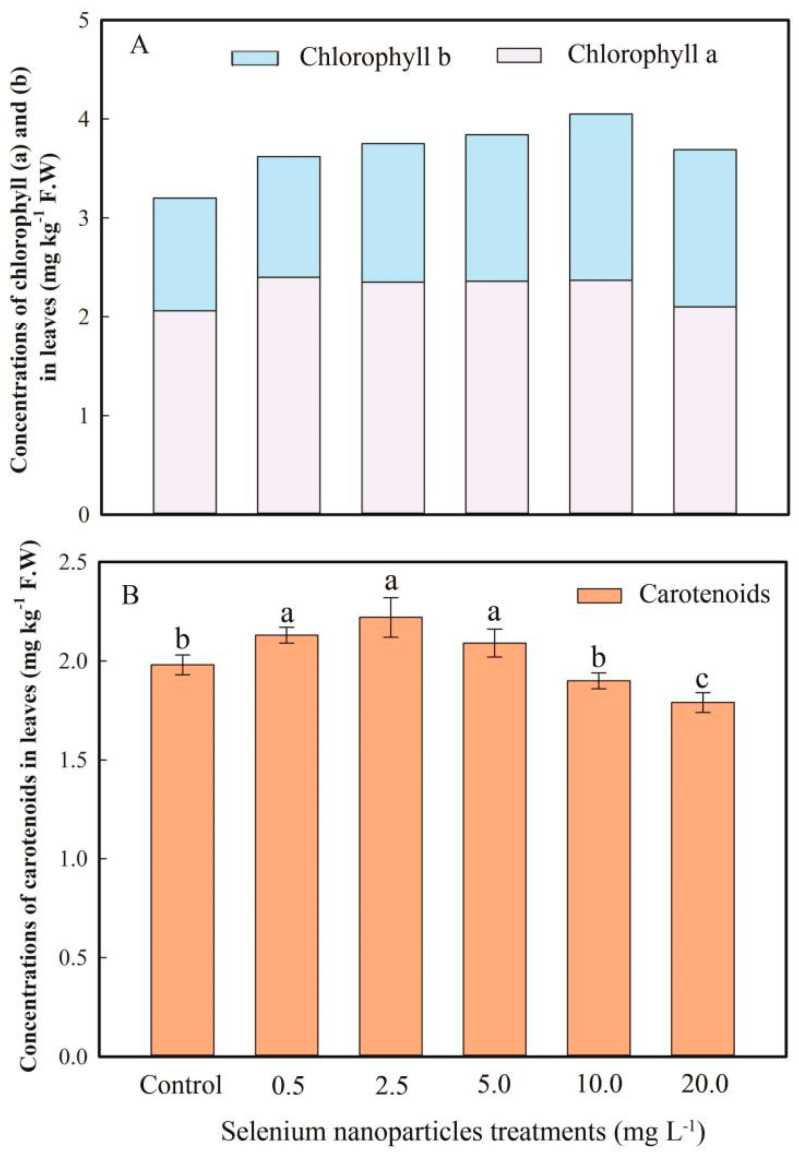
Concentrations of chlorophyll (**A**) and carotenoids (**B**) of *F. dibotrys* leaves grown under different SeNP concentrations (mg kg^−1^ F.W). Data represents mean ± SD, and means with same letter are significantly not different from each other at *p* < 0.05.

In this study, the concentrations of chlorophyll a/b were increased by SeNP treatments (Figure 5A). As shown in Figure 5, the chlorophyll a content of *F. dibotrys* leaves treated with 0.5–10.0 mg L^−1^ SeNPs was significantly increased (14.1~16.5%) compared to the control (*p* < 0.05). After application with 2.5–20.0 mg L^−1^ SeNPs, the chlorophyll b concentration increased by 12.9–35.5%. Similarly to biomass, the chlorophyll content was highest in the 5.0 mg L^−1^ and 10.0 mg L^−1^ SeNP treatments and had a significant difference compared with the control (*p* < 0.05) (Figure 5). The SeNP spray significantly increased the content of carotenoids in *F. dibotrys* leaves. However, with the 20.0 mg L^−1^ SeNP treatment, the SeNP spray significantly decreased carotenoids in *F. dibotrys* leaves (Figure 5).

### 3.6. Antioxidant Protection of SeNPs on the Leaves of F. dibotrys

**Figure 6:**
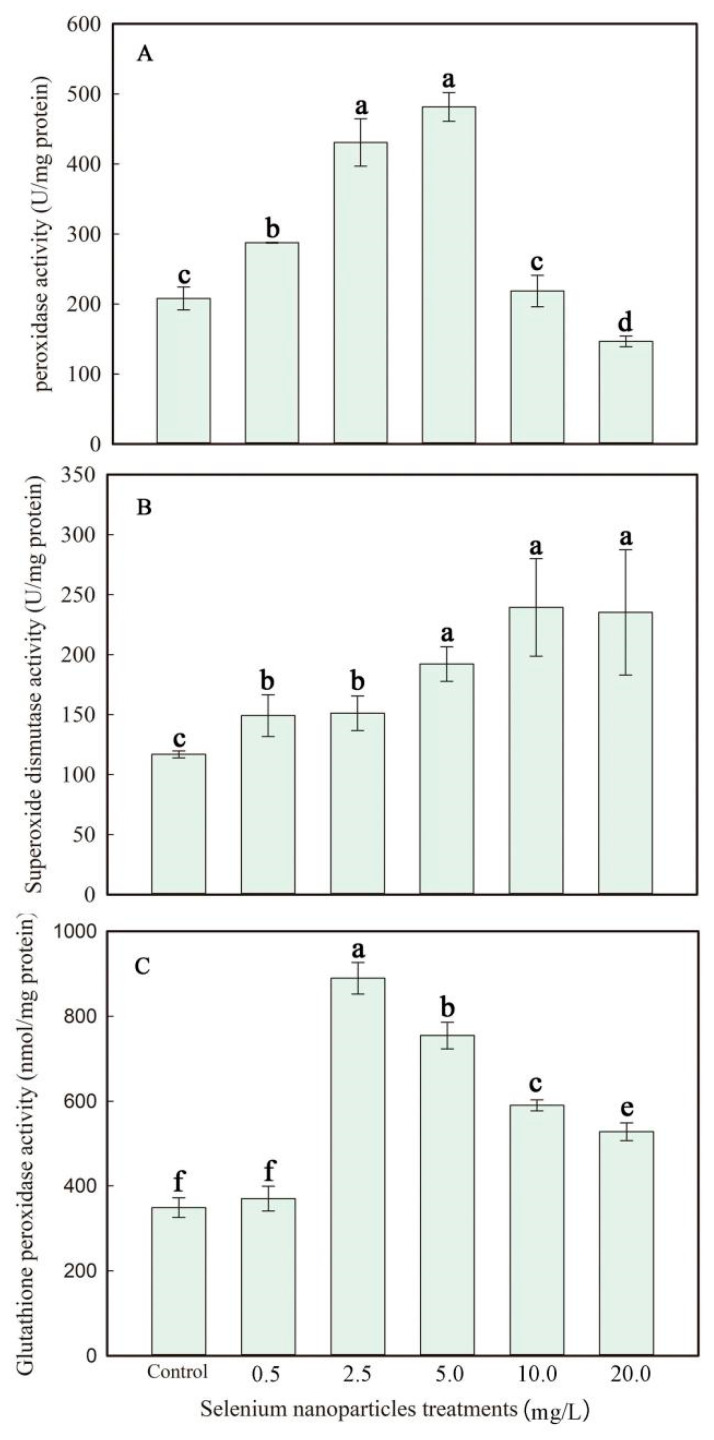
Peroxidase (POD) activity (**A**), superoxide dismutase (SOD) activity (**B**), and glutathione peroxidase (GSH-Px) activity (**C**) in the leaves of *F. dibotrys* cultivars subjected to selenium nanoparticle treatment at Se doses between 0 and 20.0 mg L^−1^. Data represents mean ± SD, and means with same letter are significantly not different from each other at *p* < 0.05.

The results showed that the POD activity in leaves increased by 38.4%, 107.2%, and 131.7% when treated with 0.5, 2.5, and 5.0 mg L^−1^ SeNPs compared to the control. However, the POD activity showed a reduction of 29.5% in the treatment with 20 mg L^−1^ SeNPs compared to the control treatment. However, in the 10.0 mg L^−1^ SeNP treatment, POD activity returned to normal levels (Figure 6). The application of SeNPs at concentrations of 0.5, 2.5, 5.0, 10.0, and 20.0 mg L^−1^ significantly raised the activity of SOD in leaves by 27.7%, 29.4%, 64.5%, 104.9%, and 101.2%, respectively, compared to the control group (Figure 6). The activity of GSH-Px in leaves was dramatically enhanced by 607.1%, 500.0%, 369.1%, and 319.8% when treated with 2.5, 5.0, 10.0, and 20.0 mg L^−1^ of SeNPs, respectively, compared to the control (Figure 6).

## 4. Discussion

In this study, pot experiments were applied to study the effects of different concentrations of SeNPs on *F. dibotrys* growth and Se accumulation. This experiment shows that the foliar application of SeNPs can increase chlorophyll a/b and carotenoid contents to promote the biomass of *F. dibotrys.* Moreover, SeNPs regulated antioxidant enzyme activities, which may attribute to improved flavonoid contents in *F. dibotrys*, especially for the SeNP treatment at 5.0 mg L^−1^ (Figure 1). Selenium concentration in leaves, stalks, and tubers of *F. dibotrys* showed an increasing trend with the application of SeNPs.

### 4.1. Accumulation and Biotransformation of SeNPs in F. dibotrys Tissues

The present study initially examined Se concentrations transferred in leaves, stalks, and tubers following foliar application. Firstly, after eight hours of spraying of SeNPs, the SEM examined the stoma of leaves, which showed no significant difference compared with the control (Figure S3). Visual analysis showed that in the 20.0 mg L^−1^ SeNP treatment, from the SEM images, the size of individual electron-dense particles was measured to be approximately 5 nm on the leaf stoma in the SeNP treatment. Nanoparticles on the leaf surface are identified by examining the surface by electron probe micro-analysis, and regional EDS analysis also showed that the nanoparticles were SeNPs (Figure S3). Stomata are pores that provide the major route for gaseous exchange across the impermeable cuticle of leaves and stems [38]. This evidence shows that *F. dibotrys* could absorb SeNPs by foliar application and transport them to other aerial parts (Figure 2). Moreover, it is justifiable to hypothesize that the uptake of SeNPs by leaf stomata during photosynthesis is not exclusively governed by the diameter of cell wall pores; other processes may also play a role in this process. Previous research found that the vacuoles have a limited capacity to store nanoparticles during apoplast transport [39]. Several studies have indicated that spherical SeNPs have the ability to penetrate plants and undergo transformation [13,14]. However, the precise locations within the plants where this transformation takes place, as well as the specific elements that govern this transformation, remain uncertain.

In this study, Se concentrations in leaves, stalks, and tubers increased steadily with the addition of SeNPs, and Se content in the leaves was higher than in the stalks and tubers (Figure 2). SeNP foliar application from 0.5 to 20.0 mg L^−1^ progressively increased the amount of Se taken up by *F. dibotrys*; 14% to 40% of Se was translocated to stalks, and 12% to 23% of Se was translocated to tubers (Figure 2). Previous studies have also shown a low translocation of Se in plants treated with SeNPs due to its ready assimilation into SeMet and several other unidentified Se species in roots [14]. Nevertheless, this research found that *F. dibotrys* leaves absorbed SeNP foliar application and its ready assimilation into SeCys_2_ (23.4 ± 1.3%) and SeMet (32.5 ± 1.5%) (Figure 3). The findings of this study indicate that *F. dibotrys* has the ability to convert ingested SeNPs into Se(IV) (40.4 ± 1.9%) and organic Se species. These results indicate that SeNPs are bioavailable for plants. The results of Se species showed that SeMet (43.2 ± 2.8%) and SeCys_2_ (38.4 ± 1.8%) were the predominant species in tubers of *F. dibotrys.* In contrast, Se(IV) (16.3 ± 1.3%) and Se(VI) (2.1 ± 0.5%) were also identified in tubers (Figure 3). The absorption process of Se(VI) in plants is metabolized via the sulfur assimilation pathway [40]. The reduction of Se(VI) to Se(IV) was facilitated by ATP sulfurylase, leading to its further conversion to SeCys by the action of selenocysteine methyltransferase [41]. Thus, the findings of this study provide evidence that the assimilated SeNPs have the potential to undergo conversion into organic Se species within plants.

### 4.2. SeNPs Regulated Growth Index and Antioxidant Enzymes

SeNPs have been used as a fertilizer source in numerous research investigations to enhance agricultural output and improve food quality [10,12]. In this study, SeNP foliar application was observed to increase the biomass of *F. diboteys* (Figure 1). According to the findings of Chauhan et al., the application of a small quantity of Se was found to have a stimulatory effect on the growth of rice plants [42]. Selenium functions as an anti-senescent agent, supporting the maintenance of cellular components and processes and enhancing plant performance [40]. However, high Se doses may inhibit photosynthetic pigment biosynthesis and suppress growth, developmental, and physiological processes [43]. Under the 20.0 mg L^−1^ SeNP foliar application, SeNPs were also attached to the leaf surface in the present study (Figure S2). Selenium was also the leading cause of toxicity, but in this study, the SeNP concentrations (0–20 mg L^−1^) had minimal or no disruptive toxicity effects on their biomass.

Chlorophyll is a vital compound that enables plants to capture sunlight for the process of photosynthesis, and its concentration has a direct impact on the rate of plant growth [44]. Therefore, it can be used as an essential indicator to measure the degree of stress in a plant due to SeNPs. When the efficiency of photosynthesis in leaves is diminished, there is a subsequent reduction in the production of carbohydrates, leading to a fall in the biomass of the shoots [45]. Se increased photosynthetic pigments in soybean (*Glycine max* L.) and *Gerbera jamesonii* [46,47]. In this research, SeNPs (0.5–20.0 mg L^−1^) also enhanced the photosynthetic properties as the concentrations of the SeNP treatments gradually increased. The shoot biomass showed the same trend (Figure 1 and Figure 5). There is an assumption that Se plays a substantial part in the process of photosynthesis, leading to an observed rise in chlorophyll concentration. The observed analysis revealed a more pronounced elevation in chlorophyll concentration compared to the corresponding increase in carotenoid levels (Figure 5). Hence, the decrease in the levels of photosynthetic pigments is considered a more responsive indicator of Se impact compared to the decrease in plant biomass.

To mitigate the occurrence of oxidative stress, plants possess the capability to engage a multitude of enzyme and non-enzyme defensive mechanisms actively [48]. SOD is an initial line of defense enzymes responsible for catalyzing the highly deadly O^2−^ conversion into the comparatively less harmful H_2_O_2_ [49]. Selenium is an elemental substance that can perform crucial roles in antioxidant systems when administered at the proper dosages [50]. SOD activity increased with the foliar application of SeNPs, indicating that SeNPs promoted the response of the *F. dibotrys* antioxidant mechanism. Selenium has been shown to positively impact plant growth by increasing the activities of antioxidant enzymes [6,51]. In this study, SeNP foliar application at 0.5 to 2.5 mg L^−1^ obviously enhanced activities of POD, SOD, and GSH-Px and increased the contents of chlorophyll and carotenoids in *F. dibotrys* (Figure 5 and Figure 6). Reports suggested that in the presence of Se, external H_2_O_2_ was primarily removed by GSH-Px [8]. Regardless of the role of GSH-Px in this study, the increasing activities of POD at 0.5 to 2.5 mg L^−1^ SeNP treatments clearly show its participation in alleviating lipid peroxidation to quench H_2_O_2_. Comparable enhancements in POD activity were similarly noted in *Trifolium repens* L. specimens subjected to selenite treatment [52]. Excess O_2_^-^ then needs more SOD to scavenge itself; in this study, the activity of SOD was significantly increased two-fold compared with the control at 10.0 to 20.0 mg L^−1^ SeNP exposure. However, the activity of POD was down-regulated at the highest SeNP exposure. The decreased activities of POD further indicate the restricted functions of POD in the tolerance of *F. dibotrys* to elevated Se levels.

### 4.3. SeNPs Increased Flavonoid Content

Flavonoids represent the most extensive category of phenolic compounds, exhibiting notable antibacterial and antioxidant properties [53]. Plant flavonoids serve multiple activities inside the plant, including protein synthesis, nutrition absorption, photosynthesis, allelopathy, enzyme catalysis, and structural support [3]. SeNP supplementation enhanced the total flavonoid content by 6.2–6.5%, 2.9–3.0%, and 2.5–3.0%, under 2.5–20.0 mg L^−1^ SeNP exposure, in leaves, stalks, and tubers, respectively (Figure 4). Notably, the total flavonoids were increased with the increase in SeNP treatment. Therefore, the SeNPs were positively correlated with producing these secondary metabolites in *F. dibotrys*. In other words, our findings confirm that the most suitable SeNP concentration for the production of flavonoids in *F. dibotrys* is 2.5–10.0 mg L^−1^ (Figure 4). Flavonoid chemicals play a crucial role in biological systems due to their significant antioxidant properties. Numerous studies have demonstrated a linear relationship between the flavonoid content and the extent of antioxidant activity [20,54]. The enhanced flavonoid compounds in *F. dibotrys* may potentially demonstrate the capacity to effectively eliminate excessive levels of ROS, hence playing a role in mitigating the toxicity associated with Se exposure. Flavonoids are synthesized through the phenylpropanoid pathway, and Se impacts the phenylpropanoid pathway; the application of Se treatments resulted in an increased carbon flow into the branched pathways of phenylpropanoid metabolism in the roots of peanut seedlings [25]. The result of flavonoid compounds being enhanced by SeNP treatments is in agreement with the previous report showing that Se regulates the phenylpropanoid pathway. Flavonoids have been recognized as one of the important classes of phytochemicals that enhance health benefits [55]. The results indicated that *F. dibotrys* enriched with Se possessed a higher concentration of flavonoids, which would enhance its medicinal properties in comparison to the control treatment. It can be inferred that Se is advantageous for *F. dibotrys,* primarily by working as an antioxidant itself and secondly by enhancing the level of another class of antioxidant, the flavonoid, during Se treatment.

## 5. Conclusions

*F. dibotrys* could absorb SeNPs through foliar application. Se was primarily accumulated in leaves rather than stalks and tubers, and it was mainly transformed into SeMet (32.5–43.2%) and SeCy_2_ (23.4–38.4%) in tissues. Exogenous SeNPs with a concentration of 5.0 mg L^−1^ promoted the growth index and increased the POD, SOD, and GSH-Px of *F. dibotrys*. This study’s results indicate that applying SeNPs to plants can enhance Se levels, improving flavonoid compounds in *F. dibotrys*. The results found in this study provided substantial evidence for further research on Se tolerance and Se accumulation in plants.

## Data Availability

The data presented in this study are available on request from the corresponding author.

## Supplementary Materials

The following supporting information can be downloaded at https://www.mdpi.com/article/10.3390/plants13213098/s1, Figure S1: Characterization of selenium nanoparticles. A TEM and B EDS analyses of nanoparticles; Figure S2: SEM images of the surface of Fagopyrum dibotrys leaves. A (1–3) is no SeNPs treatment, and B (1–3) is under 20.0 mg L^−1^ SeNPs treatment; Figure S3: Characterization of SeNPs in the surface of Fagopyrum dibotrys leaves (after 8 h of treatment). A. SEM; B. EDS analyses of NPs.
