# Supplementary material for: Selenium Nanoparticles Regulate Antioxidant Enzymes and Flavonoid Compounds in Fagopyrum dibotrys
Source: Plants (Basel). 2024 Nov 3;13(21):3098. doi: 10.3390/plants13213098 (PMC11548228; doi:10.3390/plants13213098)

Supplement Figures

Figure S1 Characterization of selenium nanoparticles. A TEM and B EDS analyses of nanoparticles.

Figure S2 SEM images of the surface of *Fagopyrum dibotrys* leaves. A (1-3) is no SeNPs treatment, and B (1-3) is under 20.0 mg L^{-1} SeNPs treatment.

Figure S3 Characterization of SeNPs in the surface of *Fagopyrum dibotrys* leaves (after 8 hours of treatment). A. SEM; B. EDS analyses of NPs.

Fig. S1

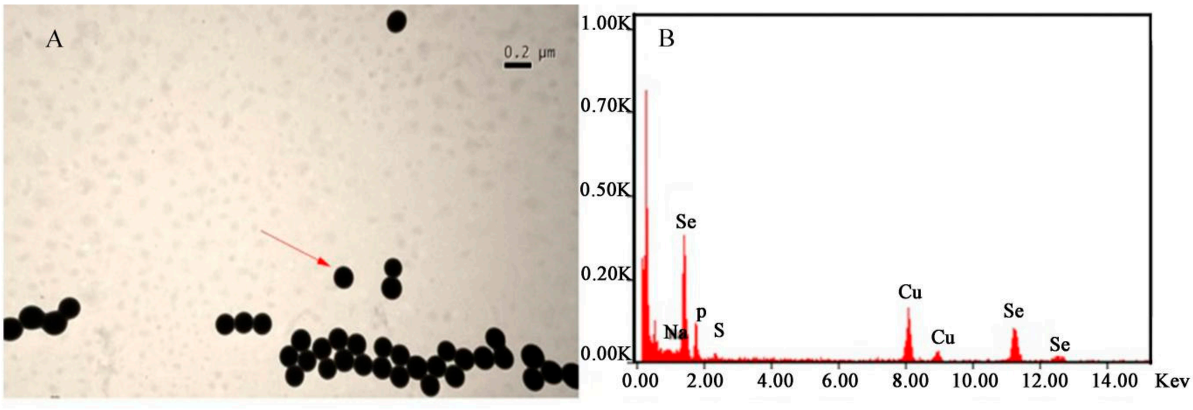

Fig. S2

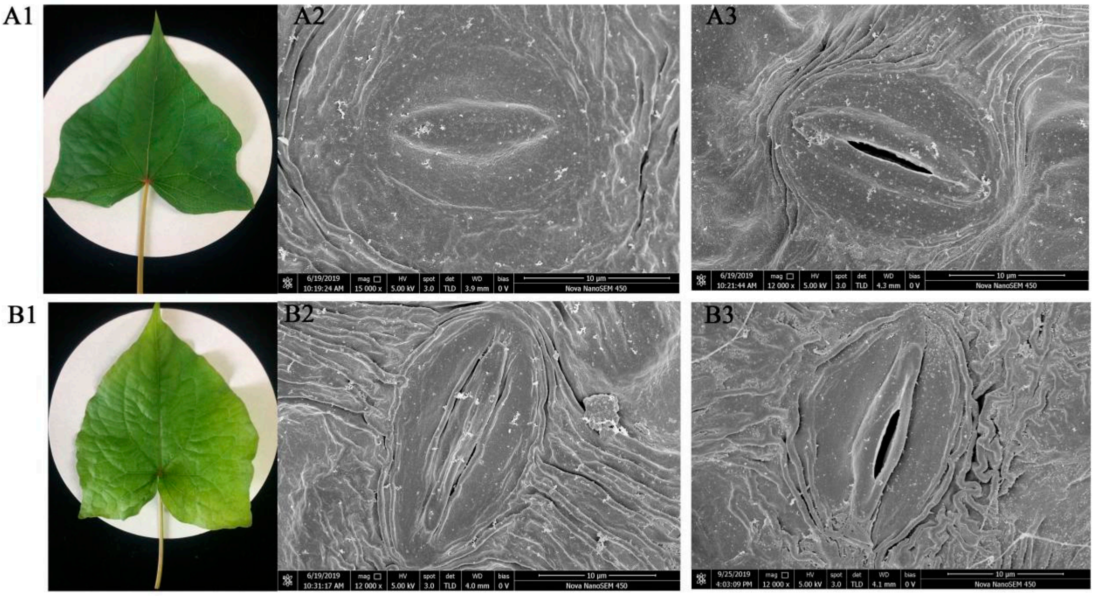

Fig. S3

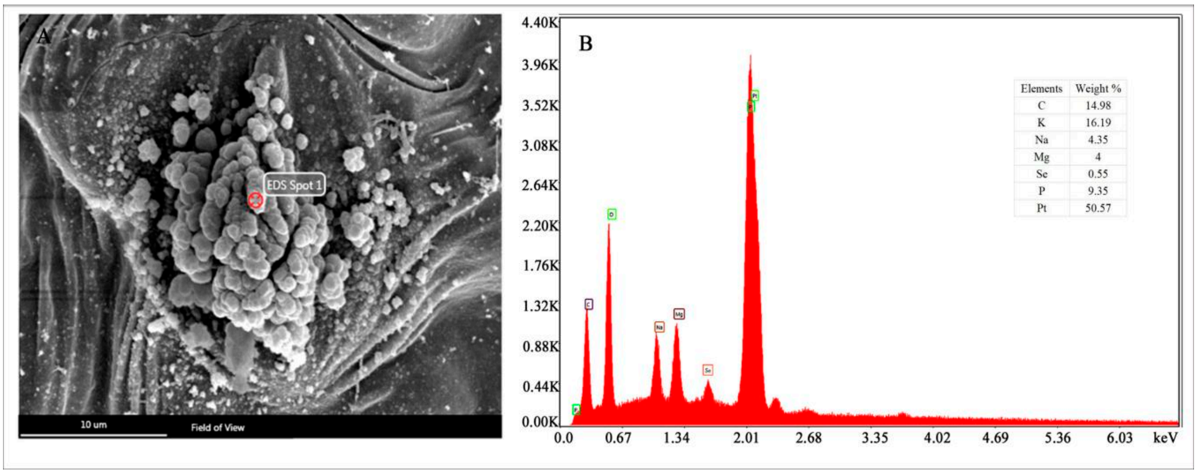

Supplement: Supplementary file 1 [file plants-13-03098-s001.zip › plants-3211844-supplementary.pdf]
